# Characterization of blood group variants in an Omani population by comparison of whole genome sequencing and serology

**DOI:** 10.1111/trf.18401

**Published:** 2025-09-07

**Authors:** Paige E. Haffener, Arwa Z. Al‐Riyami, Shoaib Al‐Zadjali, Mohammed Al‐Rawahi, Saif Al Hosni, Ali Al Marhoobi, Ammar Al Sheriyani, Ellen M. Leffler

**Affiliations:** ^1^ Department of Human Genetics The University of Utah School of Medicine Salt Lake City Utah USA; ^2^ Department of Hematology Sultan Qaboos University Hospital, University Medical City Muscat Oman; ^3^ Department of Hematology College of Medicine and Health Sciences, Sultan Qaboos University Muscat Oman; ^4^ Sultan Qaboos Comprehensive Cancer Center University Medical City Muscat Oman; ^5^ Royal Oman Police Hospital Muscat Oman

## Abstract

**Background:**

Although blood group variation was first described over a century ago, our understanding of the genetic variation affecting antigenic expression on the red blood cell surface in many populations is lacking. This deficit limits the ability to accurately type patients, especially as serological testing is not available for all described blood groups, and targeted genotyping panels may lack rare or population‐specific variants.

**Study design and methods:**

Here, we perform serological assays across 24 antigens and whole genome sequencing on 100 Omanis, a population underrepresented in genomic databases. We inferred blood group phenotypes using known genetic variants underlying antigen expression.

**Results:**

Comparison of serological to genetically inferred phenotypes resulted in an average prediction accuracy of 98.7%. By investigating 12 discordances, we describe candidate variants in the Lewis, Lutheran, MNS, and P1 blood groups that could affect antigenic expression, although further functional confirmation is required. We identify blood group alleles that, to our knowledge, have not been previously reported in Omanis, including several most common in African populations, likely introduced to Oman by gene flow over the last thousand years.

**Discussion:**

These findings highlight the need to evaluate individual populations and their population history when considering variants to include in genotype panels for blood group typing. This research will inform future genetic work in blood banks and transfusion services, ensuring that testing strategies are optimized for diverse genetic backgrounds.

**Abbreviations**
AC: allele count
AF: allele frequency
AFR: African
bp: base pairs
EUR: European
IAT: Indirect Antiglobulin Test
ME: Middle Eastern
MREC: Medical Research Ethics Committee
NS: nonsynonymous
OM: Omani
SAS: South Asian
SNVs: Single Nucleotide Variants
SQUH: Sultan Qaboos University Hospital

## 1 INTRODUCTION

In the 125 years since the discovery of the ABO blood group system, 47 different blood groups have been described in humans along with 52 associated blood group genes.^1^ Despite extensive knowledge of these various blood group systems, most have been described via case studies, and recent population genomic analyses suggest there is much left to be discovered. For instance, a study of genomic variation in African, European, South Asian, East Asian, and American populations from the 1000 Genomes project^2^ identified 1241 nonsynonymous (NS) variants within 43 blood group genes, and reported that 1000 of the NS variants (81%) were not known blood group polymorphisms, yet 357 were extracellular and thus potentially antigenic.^3^ Another study of the same dataset identified only 120 of 604 known blood group variants,^4^ with 36 of these found in at least one continental region where they had not previously been described.^4^ This suggests that many known variants affecting blood group variation are rare, and that we still lack a complete understanding of the distribution of blood group alleles in many populations.^4^

For example, a recent study from Oman demonstrated that rare, undescribed variants likely affect antigenic expression in multiple blood groups.^5^ In this study, targeted genotyping and serological assays were compared for 19 different antigens belonging to six blood group systems. Although overall concordance was high (>95%), only three antigens had 100% concordance.^5^ Discordances were likely due to the effect of genetic variants that were absent from the genotyping assay. While the prevalence of common blood group antigens or blood group alleles has been documented across much of the Arabian Peninsula,^6^, ^7^, ^8^, ^9^, ^10^, ^11^, ^12^ no comparison of sequencing data and serology has been conducted to identify additional variants affecting antigenic expression in this region.

Targeted genotyping is increasingly being investigated as an alternative or complement to serology for blood group phenotyping.^13^ Benefits of genotyping include improving red cell matching for multi‐transfused patients, those at an increased risk of alloimmunization, such as patients with sickle cell disease, and those with autoantibodies. The addition of a genotyping strategy is of interest in Oman, where hemoglobinopathies and the risk of alloimmunization are common in the population.^14^, ^15^, ^16^ However, a more complete picture of rare and population‐specific variants affecting antigenic expression is necessary. Whole genome sequencing provides a comprehensive view of blood group loci, including indels and copy number variation, particularly at more complex loci such as those that determine the Rh and MNS blood groups.^17^, ^18^ Here, we compare antigen typing inferred by whole genome sequencing to antigen expression determined by serology to identify variants contributing to blood group variation in the Omani population.

## 2 STUDY DESIGN AND METHODS

### 2.1 Sample collection

One hundred healthy male and female Omani blood donors between the ages of 18 and 60 years attending the Sultan Qaboos University Hospital (SQUH) blood bank were randomly selected and consented for enrollment in the study. The Medical Research Ethics committee at the College of Medicine and Health Sciences, the Sultan Qaboos University approved this study (MREC #2034, 2019). Additional details including DNA extraction and shipping conditions were as previously described.^19^

### 2.2 Blood Bank methods

Red blood cell phenotyping was performed within 24 h of collection at SQUH Blood bank using BioRad© antisera on freshly drawn samples as per the manufacturer's instructions (BioRad©, Cressier, Switzerland) and as previously published.^12^ The following blood systems and antigens were tested: ABO (A, B antigens), Rh (D, C, c, E, e antigens), Kell (K, k, Kp^a^, Kp^b^ antigens), Kidd (Jk^a^, Jk^b^ antigens), Duffy (Fy^a^, Fy^b^ antigens), Lewis (Le^a^, Le^b^ antigens), Lutheran (Lu^a^, Lu^b^ antigens), MNS (M, N, S, s antigens), and P1 (P_1_ antigen). A clear red cell button at the bottom of the phenotyping well was defined as a negative reaction for all antigens (grade 0). RhD reactions of 0 or 1 were further tested for weak D using the “ID‐DiaClon anti‐D” with the utilization of a monoclonal anti‐D formulated to characterize weak Ds and DVI by Indirect Antiglobulin Test (IAT) as per manufacturer's instructions. Known weak D and RhD negative samples were included with each test. Reactions positive for weak D testing were reported as Rh D positive, and reactions negative for weak D testing were reported as Rh D negative as per the manufacturer instructions. Other reaction patterns were defined as positive and were graded (1–4) for each antigen phenotyped. We included known positive and negative samples as internal controls for each antigen.

### 2.3 Genome sequencing, alignment and variant calling

As previously described,^19^ short read (150 bp paired‐end) whole genome sequencing was performed to an average coverage of 16X at the Huntsman Cancer Institute High‐Throughput Genomics Shared Resource at the University of Utah. The sequence reads were aligned to GRCh38 with BWA‐MEM^20^ and variants were called following the GATK best practices protocol.^21^, ^22^ Variants were phased using Eagle v2^23^ to produce a haplotype variant call file. A jointly called dataset, described in Haffener et al.^19^ that includes other Middle Eastern samples, was used to determine the prevalence of characterized blood group alleles in the Middle East and other populations.

### 2.4 Inferring blood group phenotypes

#### 2.4.1 ABO, RHCE, Kell, Kidd, Duffy, Lewis, Lutheran, MNS, and P1 inference with Single Nucleotide Variants (SNVs)

Using the databases available from ISBT^1^ and BloodAntigens.com,^13^ we considered genetic variants known to contribute to the expression of the tested antigens. Blood group phenotypes for each individual were predicted based on genotypes at variants segregating in the Omani population (Table 1). When multiple variants contribute to the same antigen's expression, we considered the genotypes jointly and used the inferred haplotype phase for individuals heterozygous at multiple variants. For P1, since the causal variant affecting P_1_ antigen expression remains unclear, we evaluated the antigen prediction accuracy for each of the four Single Nucleotide Variants (SNVs) that have been associated with its expression.^24^

**TABLE 1:** Blood group system and antigen prediction accuracies from the comparison of blood typing by serology and inference from known genetic variants.

Blood group	Blood group prediction accuracy^a^	Blood group antigens	Antigen frequency (% positive)	Antigen prediction accuracy^b^	Genetic variant(s) used to infer antigen expression	Allele frequency
ABO	99%	A	24%	100%	rs8176747 (p.Gly268Ala, c.803G>A)	0.095
		B	17%	99%	rs8176746 (p.Leu266Met, c.796 C>A)	0.095
					rs8176719 (p.Thr88Profs*31, c.261del)	0.21
Rh	100%	D	92%	100%	RHD*01N.01 (*RHD* deletion)	0.19
					RHD*08N.01 (notably rs748783394, 37 bp insertion)	0.0376
		C	69%	100%	*RHCE* exon 2 deletion	0.46
		c	71%	100%		
		E	23%	100%	rs609320 (p.Ala226Pro, c.676G>C)	0.12
		e	98%	100%		
					rs141398055 (p.Arg201Thr, c.602G>C)	0.015
Kell	100%	K	7%	100%	rs8176058 (p.Thr193Met, c.578C>T)	0.035
		k	100%	100%		
		Kp^a^	2%	100%	rs8176059 (p.Arg281Trp, c.841C>T)	0.01
		Kp^b^	100%	100%		
Kidd	100%	Jk^a^	84%	100%	rs1058396 (p.Asp280Asn, c.838G>A)	0.36
		Jk^b^	56%	100%		
Duffy	100%	Fy^a^	7%	100%	rs12075 (p.Gly42Asp, c.125G>A)	0.955
		Fy^b^	7%	100%		
					rs2814778 (c.‐67T>C)	0.89
					rs34599082 (p. Arg89Cys, c. 265T>C)	0.015
					rs773692057 (p.Ser62CysfsTer16, c.179_180del)	0.005
Lewis	99%	Le^a^	15%	99%	rs601338 (p.Trp154Ter, c.461G>A)	0.405
		Le^b^	69%	100%		
					rs28362459 (p.Leu20Arg, c.59T>G)	0.125
					rs812936 (p.Trp68Arg, c.202T>C)	0.725
					rs778986 (p.Thr105Met, c.314C>T)	0.735
					rs3894326 (p.Ile356Lys, c.1067T>A)	0.065
Lutheran	99%	Lu^a^	3%	100%	rs28399653 (p.Arg77His, c.230G>A)	0.015
		Lu^b^	99%	99%		
MNS	92%	M	94%	95%	rs7682260 (p.Ser20Leu, c.59C>T)	0.64
		N	59%	99%	rs7687256+rs7658293 (p.Gly24Glu, c.71G>A and c.72T>G)	0.62
		S	63%	100%	rs7683365 (p.Thr48Met, c.143C>T)	0.415
		s	81%	98%	*GYP.He(P2)* (notably rs139511876, c.270+5G>T)	0.01
					GYPB*05N.01 (*GYPB* deletion)	0.015
P1	99%	P_1_	79%	99%	rs5751348 (c.−188+3010G>T)	0.41
					rs8138197 (c.−188+2252C>T)	0.425
					rs2143918 (c.−188+2783T>G)	0.425

*Note*: Information and allele frequency of variants segregating in this dataset are shown in the last two columns.

^a^ Blood group prediction accuracy is calculated as the overall percent of correctly inferred individuals (*n* = 100) predicted by genetic variants per blood group.

^b^ Antigen prediction accuracy is calculated as the percentage of individuals with each antigen expression correctly predicted by the genetic variants.

#### 2.4.2 RHD and RHCE copy number inference

D phenotypes were inferred using a copy number analysis.^13^ Using aligned reads filtered for a MAPQ >20, coverage across the *RHD* locus (chr1:25272393–25330445) and *RHCE* locus (chr1:25360659–25430193) was calculated using SAMtools.^25^ Using the equation described by Lane et al.,^13^ a ratio of *RHD* to *RHCE* coverage between 0 and 0.5 was classified as null, 0.6 and 1.5 as hemizygous, and 1.6 and 2.5 as homozygous. RHCE C/c antigen phenotypes were also inferred using a copy number analysis suggested by Lane et al.^13^ comparing coverage of *RHCE* exon 2 to the entire *RHCE* locus for individuals homozygous for an *RHD* deletion. Since the reads should be misaligning to *RHD* exon 2, when *RHD* was present we compared the coverage of *RHCE* exon 2 to *RHD* exon 2. In individuals with no *RHD* deletion inferred, a ratio of coverage greater than or equal to 1.5 was inferred as C−c+, 0.5–1.4 as C+c+, and less than 0.5 as C+c−. In individuals inferred as hemizygous at *RHD*, we adjusted the coverage ranges to >0.67 for C−c+, 0.1–0.66 for C+c+, and <0.1 for C+c−.

#### 2.4.3 MNS copy number inference

To identify copy number variation, we inferred the underlying copy number state from observed coverage at sites with high mappability in 1600 bp windows across the *GYPA*, *GYPB*, and *GYPE* loci using a Hidden Markov Model as previously described.^19^, ^26^

#### 2.4.4 Prediction accuracy calculations

We calculated two prediction accuracies in this analysis, considering the phenotype determined by serology as truth. The blood group prediction accuracy is defined as the overall percent of all correctly inferred phenotypes from genotype data for that blood group. For instance, for the Kidd blood group, this would be calculated as follows:

$$\begin{array}{l} \frac{\left(\#\text{Correctly Inferred}\;\mathrm{as}\;\mathrm{Jk}\left(a+b-\right),\mathrm{Jk}\left(a+b+\right),\text{and}\;\mathrm{Jk}\left(a-b+\right)\mathrm{by}\;\text{Genotype}\right)}{\text{Total}\#\text{ofindividuals}\left(n=100\right)}\;\times 100\\ \;=\text{Blood Group Prediction Accuracy} \end{array}$$

The antigen prediction accuracy is calculated per antigen and is defined as the percent of individuals with individual antigen expression correctly predicted by genotype inferences over the total number of individuals. For example, prediction accuracy for the Jk^a^ antigen would be calculated as follows:

$$\begin{array}{l} \frac{\left(\#\text{Correctly Inferred}\;\mathrm{as}\;\mathrm{Jk}\left(a+\right)\;\text{and}\;\mathrm{Jk}\left(a-\right)\right)}{\text{Total}\#\text{of individauls}\left(n=100\right)}\times 100\\ \;=\mathrm{Jk}\left(a\right)\text{Antigen Prediction Accuracy} \end{array}$$

## 3 RESULTS

### 3.1 Serological findings

In 100 Omani blood donors, we applied a commonly used serology panel to phenotype 24 antigens in the ABO, Rh, Kell, Kidd, Duffy, Lewis, Lutheran, P1, and MNS blood group systems (Table S1). The frequency of antigen positivity is shown in Table 1 and the phenotype frequency in each blood group system in Table S2. The most common ABO/D phenotype was O+ (61%) followed by A+. Among the tested donors, 92% were D positive, with R1r and R1R1 being the most common presumed Rh phenotypes based on serology (29% and 28% respectively, Table S2). The frequencies of the M, N, S, and s antigens were 94%, 59%, 63%, and 81%, respectively. The Le^a^ and Le^b^ alleles were found in 15% and 69% of donors, respectively. For the Lutheran blood group system, Lu^a^ and Lu^b^ were present in 3% and 99% of the donors, respectively. The frequency of P_1_ antigen was 79%. Results for the Duffy blood group have been reported and discussed in more detail in an analysis of genomic ancestry and selection.^19^

### 3.2 Comparison of serological and genetically inferred phenotypes

To determine the genetic basis for the serological variation, we analyzed variant calls from whole genome sequencing data from the same 100 blood donors. Using genetic variants known to underlie the 24 antigens tested to infer the serological phenotype for each antigen, the prediction accuracy for each antigen ranged from 95% to 100% (Table 1). Across all blood group systems, the average blood group prediction accuracy was 98.7% (Table 1). Four blood group systems had a prediction accuracy of 100% (Rh, Kell, Kidd, and Duffy). The MNS blood group had the lowest prediction accuracy (92%). The remaining four blood groups had prediction accuracies of 99%.

### 3.3 Investigation of discordant samples

We identified a total of 12 discordances in 11 donors across five of the blood group systems (Table 2, with discordant serological results highlighted in Table S1). Nine of the discordances were false negatives, such that sequence data indicated no antigen expression but serology was positive. Three were false positives, such that sequence data indicated antigen expression but serology was negative. Candidate genetic variants that may resolve the discordance were identified in four cases and a genotyping error in one. Seven discordances remain unresolved, all of which involve the MNS blood group. We discuss the discordances and the identified variants for each blood group in detail below.

**TABLE 2:** Summary of antigen expression discordances between serology and genotype prediction (*n* = 12).

Sample ID(s)	Blood group	Phenotype by serology	Phenotype predicted by genotype	Genetic variants used for phenotype prediction and their genotypes in discordant samples	Possible explanatory variant, predicted effect, and allele count (AC)^a^
18263X61	ABO	B	O	c.803G>A: 0/1 c.796C>A: 0/1 c.261del: 0/0	NA
18263X9	Lewis	Le(a+b+)	Le(a−b+)	c.461G>A: 0/1 c.59T>G: 0/0 c.202T>C: 0/1 c.314C>T: 0/1	rs373779096 (p.Ala335Thr, c.1003G>T); AC = 1
18263X34	Lutheran	Lu(a−b−)	Lu(a−b+)	c.230G>A: 0/0	rs533045163 (c.145+6796T>A) and rs184739796 (c.145+6797C>T) altering a GATA1 binding site; AC = 1
18263X34, 18263X49, 18263X50, 18273X76, 18263X88	MNS (M/N)	M+N+	M−N+	c.59C>T: 0/0 c.71G>A: 0/0 c.72T>G: 0/0	NA
18263X32	MNS (M/N)	M+N+	M+N−	c.59C>T: 1/1 c.71G>A: 1/1 c.72T>G: 1/1	NA
18263X66	MNS (S/s)	S+s−	S+s+	c.143C>T: 0/1 altering *GYPA* and *GYPB* copy number; AC = 1	Dantu structural variant altering *GYPA* and *GYPB* copy number; AC = 1
18263X86	MNS (S/s)	S+s+	S+s−	c.143C>T: 1/1	NA
18263X75	P1	P_2_	P_1_	c.−188+3010G>T: 0/1 c.−188+2252C>T: 0/1 c.−188+2783T>G: 0/1	Chr22:42721266 (*A4GALT*, c.−289A>C) altering a STAT1 binding site; AC = 2

*Note*: Genotypes are shown for known variants influencing antigen expression that segregate in this dataset (0/0: homozygous for the reference allele; 0/1: heterozygous; 1/1: homozygous for the alternative allele). If any candidate variants were identified that may resolve the discordance, information and allele frequency information for the variants are shown. Variants are given by their coding sequence change. The first column shows the sample with the listed discordance.

^a^ AC = allele count, which indicates the number of times the alternate allele was observed among the 200 Omani haplotypes sequenced here.

#### 3.3.1 ABO blood group

One individual had discordant serological and genetically inferred phenotypes for ABO. This sample was called homozygous for the reference allele c.261delG, encoding p.Thr88Profs*31, and thereby inferred as O blood type, but expressed the B antigen via serology with a strong serological reaction (4+). Further investigation revealed that this individual had 13 reads with the reference allele (c.261delG) and 1 read with the alternate allele (c.261G). Sanger sequencing confirmed that this individual was in fact heterozygous and thus predicted as B, resolving this discordance.

#### 3.3.2 Lewis blood group

The one discordant sample in the Lewis blood group was inferred as Le(a−b+), but serologically reported as Le(a+b+), a rare phenotype indicating a functional *FUT3* allele but a weak secretor allele at *FUT2*. This individual carried a unique missense variant in *FUT2*, rs373779096 (p.Ala335Thr, c.1003G>T), not present in any other samples. Ala335Thr is located in the same exon as two other known weak secretor alleles (rs1047781 p.Ile140Phe c.418A>T and rs532253708 p.Met99Leu c.295A>C) that reduce the enzymatic activity of the encoded alpha(1,2)fucosyltransferase.^27^, ^28^, ^29^ This variant could represent a new weak secretor allele and explain the discrepancy, but further confirmation is needed.

#### 3.3.3 Lutheran blood group

The one discordant sample in the Lutheran blood group was inferred as Lu(a−b+) by genotype but serologically reported as Lu(a−b−). The presence of Lu(a−b−) is consistent with the frequency observed in the previous study in Oman,^12^ and suggests a higher frequency than elsewhere.^30^, ^31^, ^32^, ^33^, ^34^, ^35^, ^36^ The Lu(a−b−) phenotype can either be due to homozygosity for loss of function alleles or expression of the *BCAM* gene below the level of detection by serology, thought to be due to decreased expression of transcription factors regulating *BCAM* in the erythroid lineage.^35^ We looked for additional variants within the *BCAM* locus and eight erythroid transcription factors using the UCSC Genome Browser and JASPAR transcription factors tract.^35^, ^37^, ^38^, ^39^ We did not identify any loss‐of‐function alleles carried by this individual in *BCAM*. However, we did identify two adjacent SNVs falling in a GATA1 binding site for *SPI1* that are unique to this individual (rs533045163 c.145+6796T>A and rs184739796 c.145+6797C>T). These variants could represent a new allele encoding an In(Lu) phenotype and explain the discrepancy, but further evidence will be required.

#### 3.3.4 MNS blood group

Six samples were discordant for either the M or N antigen. All six expressed both antigens by serology (M+N+) but five were inferred as M–N+ and one as M+N– by genotype. Additionally, we did not identify any structural variants that could be causing these discordances.

Two samples were discordant for the S or s antigen. One was inferred by genotype as S+s+ but serologically found to be S+s−. This sample was inferred to carry the Dantu structural variant (previously identified in Haffener et al.^19^). The Dantu allele is thought to express s antigen,^40^ but weakly, and so may have been below the sensitivity of the assay here.^41^ The remaining discordant sample was inferred as S+s− but serologically found to express both antigens (S+s+). This sample does not carry any known or novel missense, nonsense, or structural variants that we could identify.

#### 3.3.5 P1 blood group

Of the four SNVs associated with P_1_ expression, phenotype prediction with rs66781836 had the lowest accuracy at 97%. The other three SNVs almost always occurred together and had a prediction accuracy of 99%, suggesting rs66781836 is less likely to be the causal variant affecting P_1_ antigen expression, consistent with previous results.^42^, ^43^ The discordant sample was heterozygous for all three SNVs and inferred by genotype as P_1_, but serologically did not express P_1_ antigen. Given that one of the three most likely causal variants, rs5751348, falls within an intronic transcription factor binding site,^43^ we investigated other transcription factor binding sites within the *A4GALT* locus. We identified a variant at c.−289A>C (chr22:42721266 in GRCh38) that falls within a STAT1 transcription factor binding site for which the discrepant individual is heterozygous and thus, if resulting in decreased expression of *A4GALT1*, could explain the absence of P_1_ expression. One other sample in this dataset was also heterozygous for this SNV, but was homozygous at the three SNVs associated with P_1_ expression. Thus, it remains plausible that this variant could affect P_1_ expression and explain the discrepancy, but further confirmation is needed.

## 4 DISCUSSION

**FIGURE 1:**
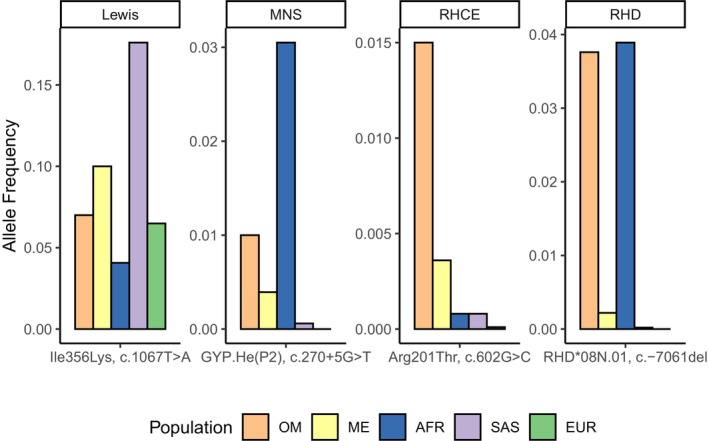
Comparison of allele frequencies of select blood group variants newly described in the Omani population (OM) with frequencies reported for Middle Eastern (ME), African (AFR), South Asian (SAS), and European (EUR) populations in gnomAD v4.1. [Color figure can be viewed at wileyonlinelibrary.com]

Here, we comprehensively document the alleles underlying common blood group antigens in an Omani population by comparison of whole genome sequencing to serology. We demonstrate high prediction accuracy for all commonly tested blood group antigens in routine transfusion practice, including 100% prediction accuracy for Rh, Kell, Kidd, and Duffy phenotypes. Achieving high prediction accuracy required inclusion of multiple recognized alleles altering blood group antigen expression that have previously not been described in Omanis. Notably, accurate prediction of D antigen phenotype required inclusion of the *RHD* pseudogene allele RHD*08N.01. This allele notably includes a 37 bp insertion in exon 4 (rs748783394) that introduces a premature stop codon resulting in early truncation of *RHD* and no expression of the D antigen.^44^ The allele frequency (AF) of RHD*08N.01 in Omanis (0.0376) is similar to the AF in African/African American population in gnomADv4.1,^45^ with AFs of 0.0376 and 0.0389, respectively (Figure 1 and Table S3). Accurate prediction of the E antigen also required inclusion of a globally uncommon weak E allele (p.Arg201Thr c.602G>C).^46^ This allele has the highest frequency in Middle Eastern populations (AF = 0.0036) in gnomADv4.1 and a frequency of 0.015 in the Omanis (Tables 1 and S3, Figure 1).

**FIGURE 2:**
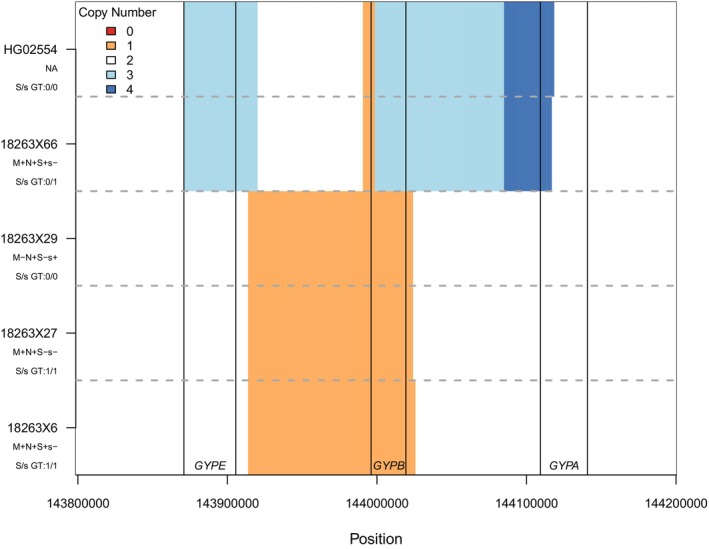
Copy number inference across the glycophorin genes based on read coverage. Position on chromosome 4 is shown on the *x*‐axis, with the locations of *GYPE, GYPB* and *GYPA* genes indicated and demarcated with black vertical lines. The inferred copy number is indicated by color for the four individuals in this dataset with deletions or duplications affecting *GYPB* or *GYPA* along with HG02554, an individual from the 1000 Genomes dataset known to carry the Dantu structural variant. Orange indicates one copy (heterozygous deletion), white indicates two copies (no copy number variant), light blue indicates three copies (heterozygous duplication), and dark blue indicates four copies (heterozygous triplication). Dotted horizontal gray lines separate the inference for each sample, with sample names indicated on the left along with the serological results for M, N, S, and s antigens and genotype for the SNV determining expression of the S or s antigen (rs7683365, p.Thr48Met, c.143C>T). [Color figure can be viewed at wileyonlinelibrary.com]

In the MNS blood group, both SNV and structural variation encode a large number of alleles, many of which are specific to African populations where the locus has been under selection likely due to the encoded proteins' roles as receptors for malaria parasites.^47^, ^48^ We identified variants at multiple sites in *GYPB* that make up the Henshaw allele *GYP.He(P2)*, which is a common cause of the S−s−U+^var^ phenotype in individuals with African ancestry^49^ (Figure 1, Table S3). Copy number calling also revealed the presence of a *GYPB* gene deletion (GYPB*05N.01 ^26^, ^50^) in three samples (Figure 2) that is common in many African populations.^26^ The notable frequency of RHD*08N.01, *GYP.He(P2)* and GYPB*05N.01 in Omanis is consistent with the shared genetic ancestry between Omani and African populations.^19^, ^51^, ^52^

In the Lewis blood group, while secretor, Le(a−b+), and non‐secretor, Le(a+b−), phenotypes are usually determined by the same nonsense mutation in *FUT2* across populations,^53^ the null phenotype, Le(a−b−), is caused by a variety of missense alleles in *FUT3* associated with reduced enzyme activity.^54^ In the 100 Omanis studied here, we observed three alleles that have been associated with Le(a−b−) in an Iranian population as well as one commonly used for genotyping the Le(a−b−) phenotype in European, South Asian, and East Asian populations (p.Ile356Lys, c.1067T>A; Figure 1, Table S3).^55^, ^56^

For each discrepancy between genotype‐based prediction and antigen expression, we searched for novel variants with a predicted functional effect and allele frequency consistent with the observed data. By this approach, we identified four variants that warrant further investigation for a possible role in antigen expression. First, within the Lewis blood group, we identified a SNV in *FUT2* that may result in the weak secretor phenotype (rs373779096, c.1003G>T, p.Ala335Thr). Presently, only two weak secretor alleles have been described (p.Ile140Phe and p.Met99Leu),^27^, ^28^, ^29^ both of which fall within the same exon of *FUT2* as the allele we identified. The identified SNV is very rare in gnomADv4.1 but primarily found in individuals of African/African American or admixed American ancestry (AF = 0.00037 and 0.00025 respectively) and in a single Middle Eastern individual (AF = 0.00017). Second, the Dantu structural variant in the MNS blood group is associated with weak s antigen expression,^40^ in this case likely below detection of the assay.

In the two individuals lacking expression of antigens despite their predicted expression using known variants, we searched for regulatory variants that could disrupt binding sites of erythroid transcription factors. In the Lutheran blood group, we report two adjacent SNVs in a GATA1 transcription factor binding site upstream of the gene encoding erythrocyte‐specific transcription factor SPI1 that could cause the In(Lu) phenotype as a result of reduced transcription of *SPI1*,^57^ akin to the SNVs in a GATA1 binding site of *EKLF* that have been associated with the In(Lu) phenotype.^35^ Although rare, these SNVs are most common to African/African American individuals in gnomADv4.1 (AF = 0.006 for both SNVs). Similarly, in the P1 blood group, we report a variant that falls within a binding site for the erythrocyte‐specific transcription factor STAT1 in the 5′UTR of *A4GALT* that may result in lack of P_1_ antigen expression. Although this variant is absent from gnomADv4.1 and dbSNP, two Omanis in this dataset were found to be heterozygous for this SNV, indicating the potential importance of including diverse populations when building blood group allele databases. We highlight that these possible links between genotype and phenotype are tentative and will require further evidence; nonetheless, they demonstrate the potential for genomic analyses to nominate new candidates to test.

While the overall blood group prediction accuracy was high, this analysis also revealed limitations to inferring some blood group phenotypes from whole genome sequencing data. The whole genome sequencing data had an average coverage of 16X and read length of 150 bp. An instance of the ABO*O.01.01 allele (c.261del, p.Thr88Profs*31) miscalled as a homozygote rather than a heterozygote suggests a deeper coverage could improve blood group inferences using commonly typed SNVs.^58^ Although the ratio of reads observed in this sample is unlikely (13 reads matching the reference allele and one read matching the alternate allele), sequencing of each chromosome is stochastic and mapping tends to be biased toward the reference allele.^59^ More encouragingly, we found that statistical phasing across multiple heterozygous genotypes in *ABO* and *FUT3* genes resulted in accurate prediction of blood group phenotypes. For example, for 14 individuals heterozygous for all three variants used to infer O and A/B, statistical phasing indicated that 13 carried the most common combination,^60^ leading to the correct prediction of B phenotype. In one individual, statistical phasing instead placed c.261del on the same haplotype as the alleles encoding ABO*B.01, resulting in the correct prediction of the A phenotype for this individual.

The high sequence homology between neighboring genes makes predicting antigen expression from short read sequence data challenging in the Rh and MNS blood group systems. In the case of the C antigen in the Rh blood group, we used a modified coverage‐based approach that improved prediction accuracy. Using the published approach from Lane et al.,^13^ which compares coverage of *RHCE* exon 2 to the rest of the *RHCE* gene, prediction accuracy for C antigen expression was 92%. Since the *RHD* exon 2 sequence present in *RHCE* in C antigen carriers is generally mismapped to the paralogous location in *RHD*, we modified this to compare read coverage at *RHD* exon 2 with *RHCE* exon 2 and prediction accuracy was 100%. However, we were unable to identify mismapped reads to improve prediction of M and N antigen expression. Correct alignment in this region of *GYPA* is particularly challenging due to the presence of the N antigen sequence in the reference at *GYPB* and the M antigen sequence in the reference at *GYPE*.^13^ We hypothesize that specific details of insert sizes and read lengths may influence whether and where allelic variation is mismapped. Long‐read sequencing or alignment to a pangenome, which can improve identification of gene conversion and structural variants,^61^, ^62^, ^63^, ^64^, ^65^, ^66^ may be necessary for consistently reliable inference of both MNS and Rh blood groups from DNA sequence.

Finally, a possible source of discordance is serology typing error. For example, two discordant samples could be due to a false‐positive serological result as the discordant antigen's serological result was weak (N and s antigens, Table S1). Repeated serological testing, particularly with alternative testing methods, would be required to determine this, but it was not possible in our study due to the lack of sufficient samples remaining.

Our findings document the alleles underlying common blood group antigens in Omanis and highlight the importance of considering population history when evaluating variants for blood group genotyping panels. Overall, this study emphasizes the necessity to increase population representation in genotype databases and indicates that whole genome sequencing paired with serology is a valuable approach for doing so in transfusion practice. Similar studies of additional populations would provide a better understanding of how prevalent red cell variants are and their relevance to red cell genotyping methods more broadly.

## AUTHOR CONTRIBUTIONS

Conceptualization—A.Z.A. and E.M.L.; Methodology—P.E.H., S.A., A.A.S., and E.M.L.; Validation—E.M.L.; Formal Analysis—P.E.H.; Investigation—P.E.H., M.A., and S.A.H.; Resources—A.Z.A. and S.A.; Writing – Original Draft, P.E.H., A.Z.A., and E.M.L.; Writing – Review and Editing, A.Z.A. and S.A.; Visualization—P.E.H.; Supervision—A.Z.A., S.A., A.A.M., and E.M.L.; Project Administration—A.Z.A.; Funding Acquisition—A.Z.A. and E.M.L. All authors have read and approved this manuscript.

## FUNDING INFORMATION

This work was supported by the Ministry of Higher Education, Research, and Innovation (formally the Research Council, grant number RC/RG‐MED/HAEM/18/02) in Oman and by the United States National Institutes of Health (grant number R35 GM147709 to E.M.L.). The computational resources used were partially funded by the NIH Shared Instrumentation Grant 1S10OD021644‐01A1. P.E.H. was supported by T32 GM007464 and T32 GM141848.

## CONFLICT OF INTEREST STATEMENT

The authors have disclosed no conflicts of interest.

## Supporting information


**TABLE S1.** Per‐sample serology results for each antigen tested. Grades 1–4 indicate a positive phenotype and expression of the antigen whereas a grade of 0 is a negative phenotype, or no expression. Each row corresponds to a sample, and each column is an individual antigen grouped by blood group system. Fields highlighted in red indicate that a discordant phenotype was inferred by genotype.
**TABLE S2:** Blood group phenotype frequencies.
**TABLE S3:** Allele frequencies of select blood group variants in Middle Eastern (ME), African (AFR), South Asian (SAS), and European (EUR) populations from gnomAD v4.1 as well as the Omani population (OM) reported here.

## Data Availability

Aligned, whole genome sequencing files (BAM) and variant calls (VCF) can be found at dbGap under accession number phs003694.v1.p1.
